# Natural Products: Therapeutic Properties and Beyond II

**DOI:** 10.3390/molecules27196140

**Published:** 2022-09-20

**Authors:** Ana Paula Duarte, Ângelo Luís, Eugenia Gallardo

**Affiliations:** 1 Centro de Investigação em Ciências da Saúde, Universidade da Beira Interior (CICS-UBI), Av. Infante D. Henrique, 6201-556 Covilhã, Portugal; 2 Laboratório de Fármaco-Toxicologia, Ubimedical, Universidade da Beira Interior, Estrada Municipal 506, 6200-284 Covilhã, Portugal

## 1. Introduction

Historically, natural products have contributed to drug discovery as a source of active molecules due to their great diversity and structural complexity. Thus, they have contributed to the development of drugs for applications in different therapeutic areas. In recent decades, there has been a paradigm shift in drug discovery strategies that has allowed for identifying new natural products that exhibit activities on therapeutic targets. Newman and Cragg studied the origin of 1330 new drugs that had been approved between 1981 and 2010 and found that 64% of them were somewhat related to natural compounds [1]. In a recent review by these same authors, it was noted that, within all of the drugs newly approved by the Food and Drug Administration between January 1981 and September 2019, compounds derived from natural products ranked second [2]. Besides the importance of the discovery of new molecules based on natural compounds, the concern today is focused on the therapeutic potential of secondary metabolites classified as drugs of abuse, such as derivatives of cannabis [3] and psilocybin [4], or even on the use of plants used ancestrally in medicine as well [5,6]. On the other hand, with the development of computational techniques, a decision has been made to study the possibilities of analyzing the pharmacological potential of natural products or their derivatives and converting these molecules into low toxicity active products. However, apart from the use of naturally occurring compounds in the field of health, they have been studied and are increasingly used in solutions, for instance in the agrochemical and food industries.

After the success of the Special Issue “Natural Products: Therapeutic Properties and Beyond I”, this second edition aims to categorize the state of the art concerning scientific research on natural products, including their applications as compounds with added value to human health. This issue intends to be used as a text for academia or as a reference tool for researchers, particularly for those working in the fields of medicinal chemistry, toxicology, phytochemistry, and natural product chemistry, and for health and industry professionals.

## 2. Contributions

This Special Issue gathers nine research papers and two reviews covering developments in the understanding of photochemical profiles, bioactivities, and safety of several natural products with applications in different fields. Concerning therapeutic properties, the anti-inflammatory, antioxidant, antifungal, antibacterial, and antidiabetic potentials of different natural compounds are addressed.

Inflammation is a physiological immune response of the body to injury, characterized by fever, swelling, and pain, and is usually implicated in the pathogenesis of a variety of diseases including, asthma, heart disease, cancer, and diabetes. In response to the increasing interest in the health-promoting effects of chaga (*Inonotus obliquus*), Alhallaf and Perkins [7] have shown that chaga collected in Maine, USA, exhibits significant anti-inflammatory properties against LPS-activated 264.7 RAW macrophages. Their results suggest that extracts produced from accelerated solvent extraction and traditional aqueous chaga tea extraction of chaga powder are superior to other conventional methods (maceration, soxhlet, and reflux) when using NO production and the expression of TNF-α, IL-6, and IL-1β as measurements of anti-inflammatory potential. 

The incidence of diabetes mellitus is increasing at an alarming rate worldwide, and the search for new drugs is currently a challenge. Feng et al. [8] studied the anti-hyperglycemic effects of refined fractions obtained from *Cyclocarya paliurus* leaves. The leaves of this plant have been widely used in ethnic medicine or herbal teas to treat diabetes in China. In recent years, the extracts of *C. paliurus* leaves were shown to reduce blood glucose and to improve insulin sensitivity on different diabetic models. These authors evaluated the chemical profiles of ethanol and the water extracts of *C. paliurus* using liquid chromatography coupled to tandem mass spectrometry and have also studied their anti-hyperglycemic effects on STZ-induced mice using glucose tolerance tests, insulin tolerance tests, and homeostasis model assessments of basal insulin resistance. The authors concluded that polysaccharides and flavonoids, but not triterpenoids, were responsible for the anti-diabetic effects of *C. paliurus* leaves. 

Baig et al. [9] analyzed the aerial parts of *Caralluma tuberculata* N. E. Br. phytochemically. It is an edible plant traditionally used in the treatment of several diseases such as dysentery, jaundice, constipation, stomach pain, freckles, and pimples. The authors observed the dependence of phytochemicals and bioactivities on the polarity of the extraction solvent. Significant responses were observed in vitro by the test extracts, namely antifungal activities, antileishmanial activities, brine shrimp cytotoxicity, THP-1 leukemia cell line cytotoxicity, and protein kinase inhibition. These findings revealed the therapeutic potential of *C. tuberculate*. 

Nazli et al. [10] explored the therapeutic potential of *Putranjiva roxburghii* through different bioactivity studies. This plant is found in Southeast Asia, and traditionally, its uses include the treatment of rheumatism, muscle twisting, fever, arthralgia, pain, and inflammation. The authors studied different parts of the plant (leaf, steam, and fruits), and apart from carrying out a phytochemical analysis, the authors stated that crude extracts are a potential reservoir of phytoconstituents, namely the considerable antioxidant, antibacterial, antidiabetic, and cytotoxic compounds that can be considered as potential candidates for the treatment of different ailments. 

An interesting study that combines in silico and experimental sets was presented by Khanzada et al. [11]. These authors explored and identified the mechanism of action of the antifungal agents of edible plants. Eight plants were selected: *Cinnamomum zeylanicum*, *Cinnamomum tamala*, *Amomum subulatum*, *Trigonella foenumgraecum*, *Mentha piperita*, *Coriandrum sativum*, *Lactuca sativa*, and *Brassica oleraceae var. italica*. These common plants were selected due to their minimal/nontoxicity, significant antioxidant and antimicrobial properties, and their frequent use in routine diet. The active phytochemicals of plants were quantified using high-performance liquid chromatography in combination with a diode array detector. In silico studies were performed to understand the underlying antifungal mechanism of detected polyphenols. The findings revealed that *C. zeylanicum*, *C. tamala*, and *A. subulatum* represented good sources of such antifungal polyphenols, and the authors suggested that the high phenolic content of the plant extracts was responsible for their antioxidant and fungal inhibitory activities. They further suggested that the polyphenols detected could be used alone or in synergy with fungal antibiotics to reduce their toxic effects and to increase antifungal efficacy. 

The study conducted by Arlotta et al. [12] aimed to evaluate the nutraceutical and genetic diversity of novel pomegranate genotypes (G1–G5) in comparison with leading commercial pomegranate varieties (Wonderful, Primosole, Dente di Cavallo, and Valenciana). The results showed that pomegranate juice is an excellent source of minerals that are essential for human health. Consuming one fruit per day may cover the daily requirement of many minerals, especially potassium, with most present in WD, DC, and G5 among the genotypes investigated. The G1–G3 and G5 genotypes presented total phenolic contents and antioxidant activities comparable with those of the commercial genotypes, representing a valid alternative to the most common cultivars for nutraceutical purposes.

Lead is a chemical toxicant that can cause severe damage to the blood and many body organs such as the liver, the kidneys, the brain, the spleen, and the lungs. It is one of the most important toxic heavy elements in the environment that can penetrate the blood–brain barrier, resulting in lead poisoning, which can cause non-traumatic brain injury. Al-Qahtani et al. [13] demonstrated that the oral administration of green tea extracts improved lead-associated pathological changes in the biochemical and neurobehavioral responses of mice treated for lead poisoning in a significant manner. 

Concerning the kinetics of natural products in the human body, Gonçalves et al. [14] evaluated in vitro the bioavailability and bioaccessibility of the main compounds present in ayahuasca beverages. Ayahuasca is a psychoactive beverage traditionally used for divine cults and medicine, but it is also known because of its recreational use. There are no studies concerning the fate of the active compounds of ayahuasca formulations after ingestion, namely concerning their absorption to general circulation for distribution. In this study, the authors observed that compounds such as *N,N*-dimethyltryptamine, harmine, harmaline, harmol, harmalol, and tetrahydroharmine were released from the matrix during the in vitro digestion process, becoming bioaccessible. Similarly, some of these compounds (except harmalol and harmol) were absorbed after incubation with the cell monolayer, becoming bioavailable.

Currently, consumers have a growing interest in substances with natural origins, such as essential oils, which have been widely used for several purposes. There has been a growing interest from different industries such as pharmaceuticals, cosmetics, and food, in using essential oils, mainly due to their biological properties, such as antifungal, antibacterial, and antioxidant activities. Ruas et al. [15] studied nationally produced (mainland Portugal and the Azores archipelago) essential oils and hydrolates obtained from forest logging and the thinning of *Eucalyptus globulus*, *Pinus pinaster*, *Pinus pinea*, and *Cryptomeria japonica*. Some of the essential oils and hydrolates showed relevant antioxidant and antimicrobial properties. Thus, it can be concluded that essential oils could be used as natural antioxidants or cosmetic preservatives, for example. Moreover, such products address the demand for sustainable and responsibly sourced odors accepted by consumers.

The review of Zuzarte et al. [16] aimed to collect and systematize relevant information on the antifungal effects of various essential oils and volatile compounds against the main types of respiratory mycoses that impact health systems (namely *Aspergillus fumigatus*, *Candida auris*, and *Cryptococcus neoformans*). The authors collected additional information on the main mechanisms of action underlying the antifungal effects of essential oils and current limitations in clinical applications. The authors stated that essential oils were rich in phenolic compounds and appeared to be very effective in respiratory mycosis, but their application in clinical practice required more comprehensive *in vivo* studies and human trials to assess efficacy and tolerability.

Orellana-Paucar et al. presented another interesting review about turmeric oil [17]. The aims of this review were to discuss its pharmacokinetics, pointing to a potential application of its active molecules in therapy. Its therapeutic potential includes antioxidant, anti-inflammatory, analgesic, antinociceptive, neuroprotective, cardiovascular, antidiabetic, nephroprotective, anticancer, antibacterial, antifungal, antiparasitic, and insecticidal properties. Most research studies reported interesting pharmacological effects without any associated toxicity. However, more studies are required to evaluate the possible clinical application of the active components of this essential oil to determine the pharmacological profile of the isolated active compounds and their bioavailability, efficacy, and safety to maximize their therapeutic benefits.

Overall, this Special Issue of *Molecules* brings together great contributions and continues to advance the discovery of natural products and the development of new applications in different areas of health sciences.
